# Settled at Last

**Published:** 1886-05

**Authors:** 


### SETTLED AT LAST.

The controversy over the place of meeting for the American Dental Association is finally ended, and the birth-place of the society, Niagara, has been chosen. This is well, and the Executive Committee is to be commended for its work. No more satisfactory conclusion could have been reached, for while some will be disappointed, no one will be offended by the choice. So much of feeling had been aroused that, had either of the places prominently mentioned been selected, there would have been found those who would have antagonized the meeting, and a split in the society might have been the result. All this is wrong, of course, but the society will not be likely again to repeat the mistakes of last year.

Now that this preliminary matter is so happily disposed of, there remains nothing except to go to work and make of the meeting what it should be. And just here it is well to consider what is meant by a successful meeting. Some will, no doubt, understand by this, one at which a great many members are present. Others would define it as a meeting that was distinguished by goodfellowship and social enjoyment. Some will judge of it by its pecuniary results, and others by the dinners eaten, the wines drunk, and the excursions or entertainments indulged in. Probably the majority of our members would not consider a meeting successful that had not a crowd in attendance. And yet, the presence of a great number of new members may actually interfere with the real, true success of a meeting, and become a thing rather to be deprecated than coveted. Understand, everyone desires the presence of all who have really at heart the best interests of dentistry. Besides, the financial question is not one to be altogether despised, and the education of the rank and file in dentistry up to an appreciation of a higher standard is a thing of vital importance. This latter consideration is, however, one that should be mainly the work of subordinate societies.

The attendance of those who come merely out of curiosity and to view the lions, to indulge in a vacation and to have a good time, to see the sights and incidentally to purchase a new stock of gum teeth and red rubber, is really not at all desirable.

The Minneapolis meeting was by many denominated a great meeting. Pray, what was there to make it such? There was a crowd in attendance, many new members were received, and the Association was royally entertained. But what great professional good has resulted? There is little left but a quarrel over the next place of meeting, in the hope that the same results might again be attained. What great discovery was there announced? What paper that marked an era in the profession was there given to the world? What remarkable discussion grew out of any presentation at that meeting? There were good papers read, and some interesting discussions grew out of them, but nothing that has made any deep impression upon the world of thought. The debates were principally over the old hackneyed subjects, and nearly the same dull round of littleness was trod. Inane trivialities were succeeded by empty platitudes, and a dreary waste of meaningless talk was too often the rule rather than the exception. Men without an original thought to present occupied valuable time, and the crude, undigested book readings of undisciplined minds were presented with an ex-cathedra air, as if the end of all research had been attained.

The American Dental Association is supposed to be a scientific society. It is intended to be a convocation of the very best intellects and intelligences in the dental profession of America. If it were what it really might be, there would be little to attract the illiterate, for the papers and discussions would be of such a technical and learned character that the average dentist could not comprehend them. They would be erudite, though not necessarily pedantical. There is, usually, very little of real scholarship in that windy rhetoric which affects high sounding phraseology, and revels in words of thundering sound. The American Dental Association, as the National society, should be above the consideration of trivial matters, and all rhetorical display should be sternly frowned down. It should devote itself to calm deliberations upon really scientific subjects, and leave the discussion of matters of detail in practice to the State and other societies with which all its members are connected. The papers presented before it should be such as to attract the attention of scientific men everywhere. As it is, with its present membership, there is an impatience manifested when anything of purely scientific interest is offered, and one who reads such an essay feels discouraged and disheartened at the coldness of its reception.

We hope there will be no misapprehension, then, when we say that a great mass-meeting of untrained minds is to be deprecated. That those meetings at which the largest numbers are present are not necessarily of the greatest moment, and that members who attend simply for the *éclat* that it may bring them, or to discuss rules of order and talk learnedly about by-laws and rules of procedure, are not those whose presence is most to be desired. That instead of dragging business questions into scientific debates, and interrupting every discussion with affairs of detail, these necessary matters should be condensed into as small compass as possible. The ideal scientific dental society will not, we fear, be attained until we hold dual meetings; one for the discussion of scientific questions, and the other for the politicians and quidnuncs whose highest ambition it is to shine in debates upon parliamentary law, and to become chronic constitution tinkers.

Shall we not at once set about the ripening of our thoughts, and each endeavor to bring to the meeting at Niagara some scientific fact, verified, not by a single instance, but by the results of a carefully studied and extended experience? Let us thoroughly prepare ourselves, and then enter into the debates, not for the purpose of gaining a polemical victory, but to discover and bring to light the truth. This would make of the meeting a grand success, no matter if but a score were present.

